# Draft Genome Sequences of Five Rapidly Growing *Mycobacterium* Species, *M. thermoresistibile*, *M. fortuitum* subsp. *acetamidolyticum*, *M. canariasense*, *M. brisbanense*, and *M. novocastrense*

**DOI:** 10.1128/genomeA.00322-16

**Published:** 2016-05-26

**Authors:** Katsuyuki Katahira, Yoshitoshi Ogura, Yasuhiro Gotoh, Tetsuya Hayashi

**Affiliations:** a Department of Bacteriology, Faculty of Medical Sciences, Kyushu University, Higashi-ku, Fukuoka, Japan; b Research Institute for Diseases of the Chest, Graduate School of Medical Sciences, Kyushu University, Higashi-ku, Fukuoka, Japan

## Abstract

We report here the draft genome sequences of five rapidly growing *Mycobacterium* (RGM) species potentially pathogenic to humans, *M. thermoresistibile*, *M. fortuitum* subsp. *acetamidolyticum*, *M. canariasense*, *M. brisbanense*, and *M. novocastrense*. As the clinical importance of RGMs is increasingly being recognized worldwide, these sequences would contribute to further advances in RGM research.

## GENOME ANNOUNCEMENT

Mycobacteria are generally classified as slow or rapid growers. Rapidly growing *Mycobacterium* (RGM) species are ubiquitous in the environment. Their clinical importance as human pathogens is being increasingly recognized worldwide (1). More than 40 species or subspecies have so far been described as RGMs. Among these, 26 are regarded as definite or potential human pathogens (1). Genome sequence information is available for 20 of the 26 (sub)species but not for 6 (sub)species. Here, we report the draft genome sequences of the following five RGM (sub)species: *M. thermoresistibile*, *M. fortuitum* subsp. *acetamidolyticum*, *M. canariasense*, *M. brisbanense*, and *M. novocastrense*. Among these bacteria, the genome sequence is available only for *M. thermoresistibile* (strain ATCC 19527^T^, accession no. AGVE00000000).

The strains that were sequenced in this study are listed in Table 1. All of them are the type strains of each species, which were obtained from the RIKEN Bio-Resource Center, and all but the *M. thermoresistibile* strain were clinical isolates (2–6). The strains were grown on Middlebrook 7H11 agar medium. Genomic DNA was extracted and purified using the ISOPLANT kit (Nippon Gene), which was used for preparing 300-bp paired-end libraries with a Nextera DNA sample preparation kit (Illumina), and sequenced by Illumina MiSeq at 40 to 80× coverage. The MiSeq reads were assembled using *Platanus* (7), yielding 70 to 140 scaffolds for each strain (Table 1). The annotation and calculation of the average nucleotide identity (ANI) were performed using the Microbial Genome Annotation Pipeline (http://www.migap.org/) and the online calculator available from EzGenome (http://www.ezbiocloud.net/ezgenome/ani), respectively.

**TABLE 1:** Summary information for the draft genome sequences of five rapidly growing *Mycobacterium* species

Species/subspecies	Strain	Source	Genome size (bp)	No. of scaffolds^a^	G+C content (%)	No. of CDSs^b^	No. of tRNAs	Accession no.
*M. thermoresistibile*	JCM6362^T^	Soil	4,893,136	85	69.0	4,716	46	BCTB00000000
*M. fortuitum* subsp. *acetamidolyticum*	JCM6368^T^	Sputum from a patient with pulmonary disease	7,101,918	83	66.0	6,981	83	BCSZ00000000
*M. canariasense*	JCM15298^T^	Blood from a patient with febrile syndrome, Spain	6,734,610	140	67.6	6,852	74	BCSY00000000
*M. brisbanense*	JCM15654^T^	Antral sinus, Australia	7,387,494	70	66.6	7,129	51	BCSX00000000
*M. novocastrense*	JCM18114^T^	Biopsy sample from slowly spreading skin granulation on a child	6,228,220	119	66.8	6,161	48	BCTA00000000

a The numbers of scaffolds >500 bp are shown.

b CDSs, coding sequences.

Similar to many of the 20 thus-far-sequenced human pathogenic RGMs, four RGMs (*M. fortuitum* subsp. *acetamidolyticum*, *M. canariasense*, *M. brisbanense*, and *M. novocastrense*) contained relatively larger genomes (6.2 to 7.4 Mb), but the genome size of *M. thermoresistibile* was relatively smaller. The sequenced *M. thermoresistibile* strain ATCC 19527^T^ also contains a small genome (4,870,742 bp), and its ANI value relative to strain JCM6362^T^ was 99.97%. The four RGMs other than *M. thermoresistibile* exhibited ANI values of <90% among them and also with all the thus-far-sequenced human pathogenic RGM species. The G+C contents of the five genomes (66.0 to 69.0%) were similar to those of the thus-far-sequenced RGMs, except for the *Mycobacterium chelonae-M. abscessus* group (63.9 to 64.1%). The numbers of protein-coding sequences that were identified in each genome were proportional to their genome sizes, but those of the tRNA genes were not proportional, as observed among the thus-far-sequenced RGMs.

Human infections by the five species sequenced here are rare, but infections in immunocompromised hosts have been reported (2–6), indicating the potential of these species as human pathogens. Their genome sequences would help further advance research on these RGM species and also fill the genome sequence information gaps on human pathogenic RGMs in the current database.

### Nucleotide sequence accession numbers.

The genome sequences described in this paper have been deposited in DDBJ/EMBL/GenBank under the accession numbers listed in Table 1. The versions described in this paper are the first versions.
